# Post-dexamethasone cortisol levels in non-functioning adrenal incidentalomas: a pooled individual participant data analysis

**DOI:** 10.1007/s12020-026-04832-z

**Published:** 2026-10-10

**Authors:** Iacopo Chiodini, Vittoria Favero, Cristina Eller-Vainicher, Valentina Morelli, Serena Palmieri, Irina Bancos, Alfredo Scillitani

**Affiliations:** 1 Department of Medical Biotechnology and Translational Medicine, University of Milan, Piazza Ospedale Maggiore 3, Milan, 20100 Italy; 2 Unit of Endocrinology, ASST Grande Ospedale Metropolitano Niguarda, Milan, Italy; 3 Unit of Endocrinology, Fondazione IRCCS Cà Granda-Ospedale Maggiore Policlinico , Milan, Italy; 4 Endocrinology Department & Lab of Endocrine and Metabolic Research, IRCCS, Istituto Auxologico Italiano, Milan, Italy; 5 Division of Endocrinology, Metabolism, and Nutrition, Department of Laboratory Medicine and Pathology, Mayo Clinic, Rochester, MN USA; 6 Unit of Endocrinology, Ospedale “Casa Sollievo della Sofferenza”, San Giovanni Rotondo, Foggia, Italy

**Keywords:** Mild autonomous cortisol secretion, Adrenal incidentalomas, Cortisol after dexamethasone suppression test, Cortisol circadian rhythm, Cortisol sensitivity

## Abstract

**Introduction:**

: Non-functioning adrenal incidentalomas (NFAI) are defined by 1 mg-dexamethasone suppression test (F-1mgDST) cortisol levels < 1.8 µg/dL. However, many individuals with NFAI may exhibit subtle hypercortisolism associated with increased cardiometabolic and skeletal risk. The aims of the present study are to compare F-1mgDST distributions between individuals with NFAI and population-based controls and to explore whether alternative F-1mgDST thresholds would meaningfully modify patient classification.

**Methods:**

This is a pooled individual participant data analysis of 7 studies evaluating cortisol secretion in patients with type 2 diabetes, osteoporosis and/or fragility fractures, hypertension, or adrenal incidentalomas. The study included 1681 participants (915 NFAI, 766 controls) with F-1mgDST < 1.8 µg/dL. Data included age, sex, body mass index (BMI), F-1mgDST, and adrenocorticotroph hormone (ACTH). We assessed the between-group differences in F-1mgDST and ACTH and the accuracy of different F-1mgDST thresholds for identifying NFAI.

**Results:**

Compared to controls, patients with NFAI had lower age and ACTH levels (62.1 ± 9.8 and 59.8 ± 12.0 years, 25.1 ± 14.1 pg/mL and 16.3 ± 10.4, respectively, *p* < 0.001 for all comparisons), but higher BMI and F-1mgDST values (28.1 ± 4.9 and 29.0 ± 5.0 kg/m^2^, 1.04 ± 0.37 µg/dL and 1.13 ± 0.39 µg/dL, respectively, *p* < 0.001 for all comparisons) and were more less frequently females (48.0% and 38.6%, respectively, *p* < 0.001). A propensity score analysis performed on 730 patients and 730 age-, BMI- and gender-matched controls confirmed these findings. We did not identify any F-1mgDST threshold with an accuracy exceeding 60% for distinguishing controls from patients with NFAI. The F-1mgDST threshold the best balance between sensitivity (54.8%) and specificity (57.2%) was set at 1.1 µg/dL (30 nmol/L), although with a low accuracy of 55.8%.

**Conclusions:**

Individuals with NFAI have higher F-1mgDST levels than controls. More than 40% overlap exists in F-1mgDST values between NFAI and controls.

**Supplementary Information:**

The online version contains supplementary material available at https://doi.org/10.1007/s12020-026-04832-z.

## Introduction

Recent evidence suggests that patients with a so-called “non-functioning” adrenal incidentaloma (NFAI), defined by post-1 mg dexamethasone suppression test (F-1mgDST) cortisol levels < 1.8 µg/dL (50 nmol/L), may nonetheless be at increased risk of cardiometabolic complications and bone fragility compared to individuals without adrenal lesions [1–3]. Importantly, in a subset of these patients, adrenalectomy led to improvement of cardiovascular risk factors [4] and resulted in postoperative hypocortisolism [5], indicating the presence of subtle autonomous cortisol secretion not detected by the F-1mgDST threshold of 1.8 µg/dL (50 nmol/L). Additionally, the increased mortality suggested in patients with NFAI [6], comparable to that reported in individuals with mild autonomous cortisol secretion (MACS) [7], supports the hypothesis that a clinically-relevant degree of hypercortisolism may be present in at least some patients classified as having NFAI. Based on these findings, the appropriateness of the F-1mgDST threshold of 1.8 µg/dL (50 nmol/L) for detecting cortisol excess is now debated [8].

The primary objectives of this study are to compare the distribution of F-1mgDST values between individuals with adrenal incidentaloma classified as NFAI and population-based controls, and to quantify the extent of overlap between groups and to assess the discriminatory performance of F-1mgDST. As a secondary exploratory objective, we sought to determine whether the application of alternative F-1mgDST.

## Design, patients and methods

This is a pooled individual analysis of all available dataset of 7 retrospective and prospective studies investigating: (i) cortisol secretion in patients with T2D [9, 10], osteoporosis and/or fragility fractures (OP/Fx) [10–12], and hypertension (AH) [10, 13]; (ii) the association of cortisol secretion with T2D, AH [14], and the prevalence and incidence of vertebral fractures in NFAI [3].

For all the examined datasets the inclusion criteria were the presence of adrenal incidentaloma (AI) incidentally detected by abdominal imaging performed for unrelated conditions and confirmed by unenhanced computed tomography (CT) a tumour size > 1 cm with well-defined tumour features consistent with adrenocortical adenoma (i.e., hypodense, homogeneous, and with Hounsfield units < 10). Patient were excluded in the presence of: (i) clinically overt cortisol excess (i.e., striae rubrae, moon facies, buffalo hump, proximal muscle weakness, and skin atrophy) and/or mild autonomous cortisol secretion (MACS; F-1mgDST levels > 1.8 µg/dL [50 nmol/L]); (ii) history of hypogonadism (in men, testosterone < 300 ng/dL [10.4 nmol/L]; in premenopausal women, < 6 menstrual cycles/year), chronic liver or kidney disease, eating disorders, thyrotoxicosis, bowel, rheumatologic or hematologic diseases, or alcoholism; (iii) use of medications affecting cortisol secretion or metabolism, or dexamethasone metabolism; (iv) bilateral adrenal masses; (v) metastatic disease; (vi) concomitant hyperaldosteronism.

A total of 1681 subjects (721 females, 42.9%) were included: 915 patients (353 females, 38.6%) with NFAI and 766 controls (368 females, 48.0%) without known adrenal disease. Controls were individuals screened for hypercortisolism in the original studies and enrolled during the same period based on the above-mentioned inclusion and exclusion criteria. All control subjects underwent F-1mgDST testing, and none had F-1mgDST > 1.8 µg/dL (50 nmol/L). They were subjects with goitre with normal thyroid function without AH, T2D and OP/FX (*n* = 283, 36.9%) or individuals with AH (*n* = 98, 12.8%), T2D (*n* = 107, 14.0%), or OP/Fx (*n* = 278, 36.3%).

Data on age, sex, body mass index (BMI), and F-1mgDST were available for all participants; ACTH levels were available for all NFAI patients and for 176 controls (23%; 99 females, 56.3%).

Statistical analyses were performed using SPSS v29.0 (IBM). Results are reported as mean ± SD, and p-values < 0.05 were considered significant. Comparisons of F-1mgDST, ACTH, age, and BMI between NFAI patients and controls were performed using Student’s t-test or the Mann-Whitney-U test, as appropriate, and gender distribution using the χ² test.

General linear modelling was used for adjusting F-1mgDST levels for age and BMI. One-way ANOVA with post-hoc LSD testing evaluated differences in F-1mgDST across control subgroups (AH, T2D, OP/Fx, or without AH/T2D).

Receiver operating characteristic (ROC) analysis assessed the association between F-1mgDST and NFAI and identified the most accurate diagnostic threshold. Sensitivity (SN), specificity (SP), positive predictive value (PPV), and negative predictive value (NPV) were calculated for F-1mgDST values from 0.6 to 1.7 µg/dL (17–47 nmol/L).

## Results

**Fig. 1:**
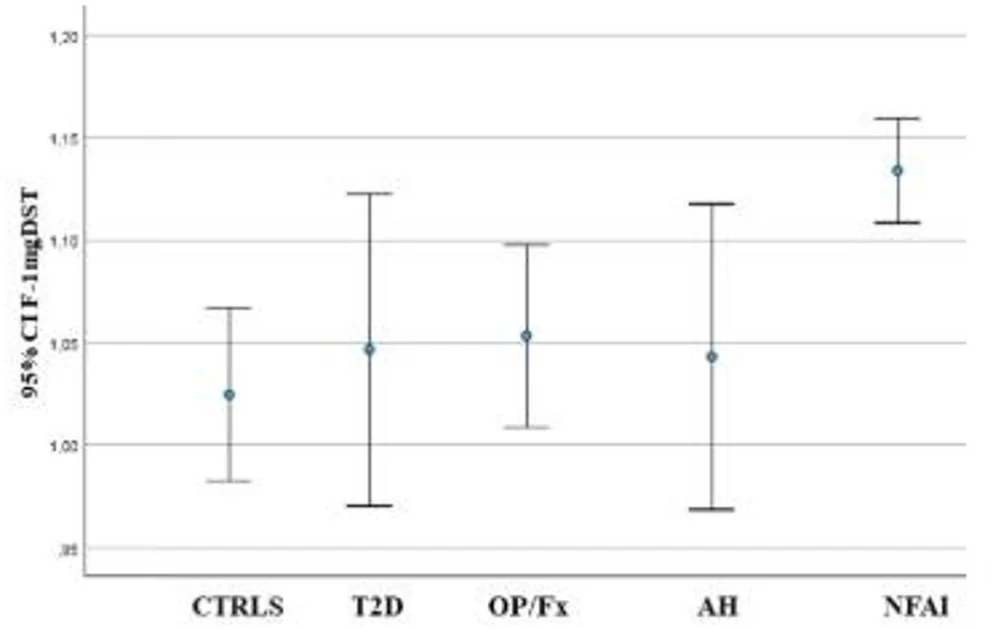
F-1mgDST levels in NFAI patients and control subjects as a whole group and divided in subgroups. Footnotes: NFAI: non-functioning adrenal incidentalomas; AH: arterial hypertension; T2D: type 2 diabetes; OP/Fx: osteoporosis and/or fragility fractures; CTRLS: healthy individuals. F-mgDST levels are expressed as mean (95%CI, Confidence Interval)

Patients with NFAI were younger and showed lower ACTH levels but higher BMI and F-1mgDST levels than controls (Table 1; Fig. 1). The F-1mgDST levels were not different across subgroups (AH, T2D, OP/Fx, or without AH/T2D) (Fig. 1).

**Table 1:** Comparison of clinical and biochemical characteristics between patients with NFAI and control individuals

Age (yrs)	NFAI (*n* = 915)	NFAI available	Controls (*n* = 766)	Controls available	*p*
	59.8 ± 12.0 (21 ‒ 89)	915	62.1 ± 9.8 (33 ‒ 85)	766	< 0.001
**Gender** (Females)	353 (38.6)	915	368 (48.0)	766	< 0.001
**BMI** (kg/m^2^)	29.0 ± 5.0 (18.3 ‒ 44.5)	915	28.1 ± 4.9 (17.5 ‒ 44.7)	766	< 0.001
**F-1mgDST** (µg/dL)	1.13 ± 0.39 (0.5 ‒ 1.79)	915	1.04 ± 0.37 (0.50 ‒ 1.79)	766	< 0.001
**ACTH** (pg/mL)	16.3 ± 10.4 (1.0 ‒ 78.0)	915	25.1 ± 14.1 (5.0 ‒ 75.0)	176	< 0.001

*Continuous variables are expressed as mean ± SD with range in parenthesis. Categorical variable are expressed as number with percentage in parentheses*

*BMI: body mass index; F-1mgDST: serum cortisol levels after 1–mg dexamethasone suppression test; ACTH: adrenocorticotroph hormone. F-1mgDST mean±standard error after adjusting for age (60.8 years and BMI 28.6 kg/m2): NFAI: 1.32 ± 0.013; controls 1.04 ± 0.014 (p < 0.001)*

*To convert to SI units multiply x: ACTH 0.22*,* cortisol 27.56*

Due to baseline clinical imbalances between the two study groups, a 1:1 Propensity Score Matching (PSM) analysis was performed to minimize potential confounding. The propensity scores were calculated using a logistic regression model based on age and body mass index (BMI), while an exact matching constraint was enforced for sex. A nearest-neighbour matching algorithm without replacement was applied, yielding a final balanced cohort of 730 patients with NFAT and 730 controls. Post-matching balance was rigorously verified using Standardized Mean Differences (SMDs < 0.1) and p-values, confirming no statistically significant differences between the matched groups for age (*p* = 0.489), BMI (*p* = 0.204), and sex (*p* = 1.000).

By ROC curve, F-1mgDST threshold of 1.1 µg/dL (30 nmol/L) had the best compromise between SN and SP (54.8% and 57.2%, respectively, *p* < 0.001, area under the curve 0.571, SE 0.014, 95%CI 0.543–0.598) for individuating patients with NFAI. No F-1mgDST threshold showed an accuracy exceeding 60% for distinguishing controls from patients with NFAI (supplementary table). Optimal cut-offs and diagnostic performance were similar in women and men (data not shown). The exclusion of 6 patients with ACTH concentrations above the upper limit of the reference range of our assay (> 55 pg/mL) did not materially change the results of the analysis (data not shown). Finally, F-1mgDST levels showed no discriminatory ability for the presence of OP/Fx in combination with at least one cardiometabolic comorbidity (T2D, AH or OB, AUC 0.486 ± 0.023, *p* = 0.544).

## Discussion

The reliability of the F-1mgDST threshold of 1.8 µg/dL (50 nmol/L) has recently been questioned, as some patients with NFAI and lower cortisol levels may remain at risk of cortisol-related complications [8]. The primary objectives were to compare F-1mgDST values between NFAI patients and controls and quantify their overlap. As a secondary exploratory objective, we assessed whether alternative F-1mgDST thresholds resulted in clinically meaningful changes in patient classification. In this pooled analysis of 1681 participants, NFAI patients had higher F-1mgDST and lower age and ACTH levels than controls. Because controls were derived from clinical populations enriched for conditions potentially associated with cortisol excess (i.e. AH, T2D, OP/Fx), their age distribution may have been skewed toward older individuals. However, these results were further confirmed by propensity score analysis, which enabled the matching of patients and controls according to age, gender, and BMI. Although the F-1mgDST threshold of 1.1 µg/dL (30 nmol/L) had the best compromise between SN and SP for individuating patients with NFAI, we observed an overlap exceeding 40% in F-1mgDST values between patients with NFAI and controls and no F-1mgDST threshold provided adequate discriminatory performance, with accuracy remaining below 60% across all tested cut-offs.

The observation that F-1mgDST levels are higher and ACTH levels lower in NFAI patients compared with controls suggests that the commonly used threshold of 1.8 µg/dL (50 nmol/L) may not reliably exclude MACS. These finding challenges current guidelines for the management of adrenal incidentalomas [15], which consider NFAI patients with F-1mgDST < 1.8 µg/dL at low risk for cortisol-related complications. These results are consistent with data showing that at least a subgroup of NFAI patients have an increased risk of cardiometabolic complications and bone fragility compared with individuals without adrenal lesions [1–3].

However, the substantial (> 40%) overlap in F-1mgDST values between patients with NFAI and controls, together with the absence of any threshold achieving an accuracy greater than 60%, underscores the limited discriminatory performance of F-1mgDST when used in isolation and raises concerns about its reliability as a standalone tool for risk stratification in this setting. Indeed, although a threshold of approximately 1.1 µg/dL (30 nmol/L) showed the best balance between sensitivity and specificity, its poor diagnostic performance indicates that lowering the F-1mgDST threshold alone would not provide reliable discrimination between NFAI and controls. Moreover, F-1mgDST levels did not show discriminatory ability for the coexistence of bone disease and cardiometabolic comorbidities in our cohort. Although this finding should be interpreted cautiously given the composite nature of the endpoint, it further suggests that lowering the F-1mgDST threshold alone may not improve the identification of patients with a clinically relevant burden of cortisol-related comorbidities.

Importantly, we should be aware that lowering the cut-off could result in diagnosing more individuals with hypercortisolism even when they do not have the disorder. Therefore, any adjustment to the F-1 mg DST cut-off should be validated through prospective studies, ensuring that the gains in sensitivity outweigh the risks associated with false-positive results [8]. In NFAI, ACTH levels do not appear to provide additional clinically relevant information. Although lower, ACTH levels are not suppressed, which may reflect HPA axis hyperactivity, a common finding in the general population, particularly in the context of severe obesity, mental health disorders, uncontrolled T2D, and alcohol abuse. Importantly, excluding patients with ACTH > 55 pg/mL did not modify our findings, but this does not support 55 pg/mL as a reliable cut-off for defining ACTH-independent cortisol secretion. Overall, ACTH levels alone may not reliably distinguish ACTH-independent from ACTH-dependent cortisol secretion in NFAI [16].

This study is a pooled individual participant data analysis including both retrospective and prospective cohorts. Limitations include the lack of dexamethasone levels measurement, the heterogeneous design, the inclusion of control subjects with AH, T2D, and OP/FX who were screened for hypercortisolism, and the lack of a control group with confirmed absence of adrenal lesions. As controls were derived from populations screened for cortisol-related comorbidities, some may have had NFAI; however, this would likely have strengthened rather than weakened our findings. Finally, we cannot exclude confounding from different cortisol assays, potential false-positive results not ruled out by dexamethasone measurement, or non-neoplastic hypercortisolism.

In conclusion, the present individual-participant pooled analysis specifically addresses the distribution and discriminatory performance of F-1mgDST in patients with NFAI compared with subjects without known adrenal disease. Although a threshold of 1.1 µg/dL (30 nmol/L) showed the best balance between sensitivity and specificity, its discriminatory performance was poor, indicating that no F-1mgDST threshold tested could reliably distinguish NFAI patients from controls. Notwithstanding the limitations of this study, these data may serve as a foundation for future investigations.

## Supplementary Information

Below is the link to the electronic supplementary material.

Supplementary Material 1

## Data Availability

The data are available upon request.
