# Supplementary material for: Post-dexamethasone cortisol levels in non-functioning adrenal incidentalomas: a pooled individual participant data analysis
Source: Endocrine. 2026 Oct 10;91(1):344. doi: 10.1007/s12020-026-04832-z (PMC13653952; doi:10.1007/s12020-026-04832-z)
Supplement: Supplementary file 1 [file 12020_2026_4832_MOESM1_ESM.docx]

**Supplementary table.** Sensitivity, specificity, positive predictive value, negative predictive value and overall accuracy of different threshold of F-1mgDST in discriminating between NFAI patients and individuals without NFAI

| **F-1mgDST cut-off** | **SN** | **SP** | **NPV** | **PPV** | **ACC** | **p** |
| --- | --- | --- | --- | --- | --- | --- |
| 0.6 µg/dL | 89.3 | 13.8 | 55.3 | 52.0 | 54.9 | 0.05 |
| 0.7 µg/dL | 85.2 | 19.7 | 55.9 | 52.8 | 55.3 | 0.007 |
| 0.8 µg/dL | 79.1 | 28.2 | 56.8 | 53.1 | 55.9 | 0.001 |
| 0.9 µg/dL | 71.8 | 35.2 | 57.0 | 51.1 | 55.1 | 0.002 |
| 1.0 µg/dL | 63.0 | 46.1 | 58.2 | 51.0 | 55.2 | 0.001 |
| 1.1 µg/dL | 54.8 | 57.2 | 60.4 | 51.4 | 55.8 | 0.001 |
| 1.2 µg/dL | 45.4 | 66.8 | 62.0 | 50.6 | 55.1 | 0.001 |
| 1.3 µg/dL | 39.6 | 73.0 | 63.6 | 50.3 | 54.7 | 0.001 |
| 1.4 µg/dL | 31.4 | 79.4 | 64.5 | 49.2 | 53.2 | 0.001 |
| 1.5 µg/dL | 13.6 | 85.0 | 66.5 | 48.7 | 52.3 | 0.001 |
| 1.6 µg/dL | 10.2 | 89.4 | 67.9 | 47.9 | 50.9 | 0.001 |
| 1.7 µg/dL | 6.4 | 93.5 | 68.4 | 47.0 | 49.0 | 0.001 |

F-1mgDST: serum cortisol levels after 1–mg dexamethasone suppression test. SN: sensitivity, SP: specificity; NPV: negative predictive value; PPV: positive predictive value; ACC: accuracy
